# Changes in cardiac function and hemodynamics during robot-assisted laparoscopic prostatectomy with steep head-down tilt: a prospective observational study

**DOI:** 10.1186/s13104-017-2672-z

**Published:** 2017-07-28

**Authors:** Naomi Ono, Junko Nakahira, Shoko Nakano, Toshiyuki Sawai, Toshiaki Minami

**Affiliations:** Department of Anesthesiology, Osaka Medical College, 2-7 Daigaku-machi, Takatsuki, Osaka, 569-8686 Japan

**Keywords:** Robot-assisted laparoscopic prostatectomy, Steep head-down tilt, Transesophageal echocardiography

## Abstract

**Objective:**

Robot-assisted laparoscopic prostatectomy requires the patient to be placed in a steep head-down tilt. The aim of our study was to investigate changes in cardiac index and left ventricular end-diastolic volume in a steep had-down tilt. This is a prospective observational study. We investigated the influence of steep head-down tilt on cardiac function and hemodynamics without fluid restriction in 12 men of American Society of Anesthesiologists physical status I–II undergoing robot-assisted laparoscopic prostatectomy. We measured left ventricular ejection fraction, left ventricular end-diastolic volume and cardiac index by transesophageal echocardiography, cardiac index using a FloTrac^®^ sensor, heart rate and arterial blood pressure, before and 5 min after tilting the operating table.

**Results:**

The following variables changed significantly after tilting and establishment of the pneumoperitoneum: left ventricular ejection fraction (before 62.5%, after 55.5%; P = 0.040), systolic blood pressure (before 116 mmHg, after 128 mmHg; P = 0.001) and diastolic blood pressure (before 59 mmHg, after 70 mmHg; P = 0.002). There were no significant changes in cardiac index or left ventricular end-diastolic volume measured by transesophageal echocardiography, or cardiac index by FloTrac^®^ sensor. Left ventricular ejection fraction decreased, whereas cardiac index and left ventricular end-diastolic volume did not change, indicating that steep head-down tilt and pneumoperitoneum during robot-assisted laparoscopic prostatectomy did not greatly influence cardiac function.

This study was registered as a clinical study with the Japanese Official Clinical Trial Registry (Trial Registration Number JMA-IIA00158 on 7th January, 2014)

## Introduction

Robot-assisted techniques have gained popularity because they overcome several of the shortcomings of conventional laparoscopic techniques. The problems associated with robotic prostatectomy are a consequence of four main factors: the steep Trendelenburg position of the patient, the insufflation of carbon dioxide to produce a pneumoperitoneum, spatial restrictions because of the bulk of the equipment set over the patient, and the possibility of unexpected visceral injury or blood loss [1].

A few cases of cardiopulmonary deterioration, evidenced by pulmonary edema, pulmonary embolism and worsening of mitral regurgitation, have been reported during this procedure [2–4]. We investigated the changes in hemodynamic parameters and cardiac function during robot-assisted laparoscopic prostatectomy (RALP) without fluid restriction.

## Main text

### Methods

The study protocol was approved by the Ethics Committee of Osaka Medical College (Reference Number 1339), and all participants provided written informed consent. This study was registered as a clinical study with the Japanese Official Clinical Trial Registry (Trial Registration Number JMA-IIA00158). Twelve men scheduled for RALP under general anesthesia were assessed in this prospective observational study. All subjects were selected after routine screening and all met the criteria for American Society of Anesthesiologists (ASA) physical status classification I–II. Routine preoperative evaluation included electrocardiography, chest radiographs, hematocrit and a screening chemistry panel to aid in identifying underlying myocardial ischemia, chronic obstructive pulmonary disease, anemia and hyperglycemia. Patients with cardiac disease, including dysrhythmia and coronary artery disease, and those with moderate or severe obstructive pulmonary disease, did not meet the criteria for this study. All RALP procedures were performed on the same ALPHAMAXX^®^ operating table, (MAQUET Holding B.V. and Co. KG, Rastatt, Germany) and with the *da Vinci* ^*®*^ Surgical System (Intuitive Surgical, Sunnyvale, CA, USA).

No premedication was given. Anesthesia was induced with propofol 1.5–2.0 mg/kg, inhaled sevoflurane 3.0% and remifentanil 0.3–0.5 µg/kg/min, and maintained with remifentanil 0.1–0.3 μg/kg/min and inhaled sevoflurane 1.5%. Rocuronium 0.5 mg/kg was used to facilitate orotracheal intubation and was repeated as needed for muscle relaxation. The patients were intubated with a cuffed endotracheal tube and their lungs mechanically ventilated in volume-controlled mode with a mixture of oxygen and air: the fraction of inspired oxygen was 0.4, the tidal volume was 7 mL/kg predicted ideal body weight and positive end-expiratory pressure (PEEP) of 5 cm H_2_O was provided. After induction of anesthesia, ventilation was adjusted to achieve an end-tidal carbon dioxide tension of 32–36 mmHg. If oxygen saturation measured by pulse oximetry was less than 97%, the tidal volume was increased to 8.5 mL/kg, and the PEEP increased. No changes were made in ventilator settings between before- and post-tilting measurements. At least 1000 mL of intravenous crystalloid or colloid was infused before the patient was placed in the steep head-down tilt.

Patients’ clinical records were reviewed and relevant patient background information, including age, body mass index and intraoperative variables, were collected. Pre-tilting measurements were performed under stable anesthesia and steady-state conditions with the patients in the horizontal position before surgery began. Post-tilting variables were measured 5 min after the patients had been positioned in a 28° head-down tilt and a pneumoperitoneum of 12–15 mmHg had been achieved. Measured variables comprised left ventricular end-diastolic volume (LVEDV), left ventricular ejection fraction (LVEF) and cardiac index (CI) by transesophageal echocardiography (TEE), blood pressure (BP) in the radial artery, CI using a FloTrac^®^ sensor and Vigileo^®^ monitor (Edwards Lifesciences, Irvine, CA, USA), central venous blood oxygen saturation (ScvO_2_) using a PreSep^®^ central venous oximetry catheter (Edwards Lifesciences), and heart rate and regional hemoglobin oxygen saturation (rSO_2_) using an INVOS™ 5100C cerebral/somatic oximeter (Medtronic Minimally Invasive Therapies, Minneapolis, MN, USA). A multiplane TEE probe, iE33^®^ X7-2t transducer (Philips N.V., Amsterdam, the Netherlands) was introduced into the esophagus after induction of anesthesia. The LVEF was calculated in the mid-esophageal four-chamber view, whereas the left ventricular end-diastolic and end-systolic areas were measuring using an iE33 ultrasound system using the modified Simpson’s method (Philips). Urine output was determined by insertion of a urinary catheter during the surgical procedure and a proportion of the urine was included in the total blood loss.

Preliminary data for LVEDV recorded in a pilot study of nine patients undergoing RALP were 125.8 ± 25.2 mL (mean ± standard deviation [SD]) before tilting and 116.6 ± 27.4 mL after tilting. The SD was therefore considered to be 25.2 and the expected difference in LVEDV 10 mL. Sample size calculation informed by these data, with an α error level of 5% and an expected pre-and post-tilting difference in LVEDV of 10 mL, found that 60 subjects would be required to obtain an 80% power goal for comparing pre- and post-head down tilt. However, the study was stopped prematurely after 12 subjects had been enrolled as the researchers moved to the different institutes. The pre- and post-tilting measurements obtained were compared using Wilcoxon signed-rank tests, with P < 0.05 considered statistically significant. Data are presented as medians with interquartile ranges. All statistical analyses were performed using GraphPad Prism 6 software (GraphPad Software, La Jolla, CA, USA).

### Results

We obtained data from 12 patients. There were no episodes of severe bradycardia. A crystalloid was infused in all cases and an additional 500 mL of colloid was infused in three cases. There were no instances of uncontrollable hemorrhage or respiratory disorder intra- or post-operatively.

Relevant patient characteristics and intraoperative variables are shown in Table 1. Comparison of measured variables before and after tilting and establishment of the pneumoperitoneum showed significant changes in median LVEF (before 62.5%, after 55.5%; P = 0.040), systolic BP (before 116 mmHg, after 128 mmHg; P = 0.001), diastolic BP (before 59 mmHg, after 70 mmHg; P = 0.002) and rSO_2_ (left: before 64%, after 67%, P = 0.034; right: before 65%, after 70%, P = 0.003) (Table 2). There were no significant changes in median LVEDV measured by TEE (before 124.0 mL, after 119.5 mL; P = 0.955), CI measured by TEE (before 2.5 L/min/m^2^, after 2.2 L/min/m^2^; P = 0.143), CI by FloTrac^®^ sensor (before 2.3 L/min/m^2^, after 2.3 L/min/m^2^, P = 0.350) or SvcO_2_ (before 83%, after 85%, P = 0.200).

**Table 1:** Relevant patient characteristics and intraoperative variables

Variable	Data, n = 12 (interquartile range)
Patient background	
Age (years)	67 (65–68)
Height (cm)	165.3 (160.3–168.8)
Body weight (kg)	71.6 (64.3–76.7)
Body surface area (m^2^)	1.78 (1.70–1.85)
Body mass index (kg/m^2^)	26.2 (23.6–28.5)
Intraoperative variables	
Anesthetic time (min)	304.0 (290.8–311.5)
Operation time (min)	204.5 (195.5–234.0)
Pneumoperitoneum time (min)	173.0 (152.0–185.0)
Volume infused (mL)	2750 (2175–3375)
Volume of urine (mL)	105 (40–298)
Volume of blood loss (mL)	200 (100–587.5)

Data are expressed as medians with interquartile ranges

**Table 2:** Comparison of circulatory variables

Variable	Pre-tilting (range)	Post-tilting (range)	P value
Basic hemodynamic variables			
Heart rate (/min)	60.0 (56.3–64.5)	59.0 (53.3–64.5)	0.148
Systolic BP (mmHg)	116.0 (101.3–125.0)	127.5 (110.0–130.0)	0.001
Diastolic BP (mmHg)	59.0 (55.0–72.5)	70.0 (61.0–80.0)	0.002
ET-CO_2_ (mmHg)	36.0 (33.3–38.5)	37.5 (34.3–38.8)	0.080
SpO_2_ (%)	100 (98.5–100)	99.5 (99.0–100)	0.625
FloTrac^®^ sensor data			
Cardiac index (L/min/m^2^)	2.28 (2.17–2.55)	2.30 (2.20–2.85)	0.350
TEE data			
Cardiac output (L/min)	4.3 (3.8–5.1)	4.0 (3.1–4.7)	0.129
Cardiac index (L/min/m^2^)	2.5 (2.2–3.0)	2.2 (1.7–2.8)	0.143
Ejection fraction (%)	62.5 (57.0–67.3)	55.5 (50.5–60.8)	0.040
Stroke volume (mL)	75.0 (61.0–89.5)	79.0 (59.3–87.0)	0.235
PreSep^®^ catheter data			
ScvO_2_ (%)	83.0 (78.3–86.8)	85.0 (79.5–87.5)	0.195
INVOS™ oximeter data			
Left rSO_2_ (%)	64.0 (59.8–75.5)	66.5 (61.8–79.3)	0.034
Right rSO_2_ (%)	64.5 (61.8–68.5)	69.5 (64.8–73.5)	0.003

Data are expressed as medians with interquartile ranges

*ET*-*CO*_*2*_ end-tidal carbon dioxide, *LVEDV* left ventricular end-diastolic volume, *rSO*_*2*_ regional saturation of oxygen, *SpO*_*2*_ percutaneous oxygen saturation, *ScvO*_*2*_ central venous blood oxygen saturation

### Discussion

We examined changes in circulatory status by measuring hemodynamic and cardiac function brought about by 28° head-down tilting and establishment of the pneumoperitoneum in men undergoing RALP. We found that head-down tilt and pneumoperitoneum significantly decreased LVEF but that LVEDV and CI measured by TEE, and CI measured with a FloTrac^®^ sensor, did not change. These findings indicate that a steep head-down tilt and pneumoperitoneum during RALP did not greatly influence cardiac function in our study patients.

Augmented venous return may result in an increase in right ventricular filling. The high inspiratory pressures required for mechanical ventilation following positioning of a patient in a steep head-down tilt and creation of a pneumoperitoneum reportedly increase right ventricular afterload, reducing right ventricular ejection and consequently increasing right ventricular volumes [2]. A steep Trendelenburg position and pneumoperitoneum may also increase left ventricular afterload because blood flow to the left ventricle is augmented by the increase in right ventricular output. Pulmonary artery wedge pressure is reportedly more than twofold that of initial values [5]. Although left ventricular end-diastolic pressure (LVEDP) provides the most useful information concerning left ventricular afterload, we did not measure this in our study. We found no significant changes in cardiac output (CO) or CI measured by TEE and FloTrac^®^ sensing; both these variables reflect stroke volume and heart rate, neither of which changed. Ejection fraction is determined by LVEDV and left ventricular end-systolic volume. Although stroke volume and LVEDV did not change, LVEF decreased in our cohort, suggesting that the decrease in LVEF was not clinically significant. Rosendal et al. reportedly identified no changes in cardiac contractility throughout RALP procedures, although the afterload increased more than twofold [6, 7]. Several studies have reported values for CO and CI after steep head-down tilting for RALP, CI having been reported differently in different studies. Haas et al. reported an increase in CO, Lester et al. and Rosendal et al. reported no changes in CI, whereas Darlong et al., Danic et al. and Felabella et al. all reported decreases in CI [2, 6, 8, 9]. Age, cardiovascular status, obesity and volume status may influence these variables [1]. According to a study in which a fluid challenge was administered, arterial elastance is an important influence on mean arterial pressure (MAP) response to head-down tilting and creation of a pneumoperitoneum [10]. We did not restrict fluids in our study, nor did we measure LVEDP, which is an important variable. Given that we did not restrict volume of infused fluids, a high LVEDP may have helped us to identify fluid overloading. We were unable to measure LVEDP or left atrial pressure in our patients as we did not catheterize the pulmonary artery, a substantially more invasive means of measuring CO and CI. We also did not measure pulmonary venous flow velocity pattern to make assessments of changes in LVEDP.

Several authors have recommended fluid restriction during radical prostatectomy to facilitate adequate visualization of the surgical field and minimize blood loss, because this procedure may induce adverse respiratory, cardiovascular, and neurophysiological changes such as severe laryngeal edema, respiratory complications and brachial plexus injury [10, 11]. In contrast, some surgeons have insisted that restricted fluid management (defined as <2000 mL for each case) causes postoperative oliguria [12], and the surgeons in our institute judge that intra- or postoperative hypovolemia may result in more serious adverse events than an obscured operative field during bladder neck transection. The policy of our institution is therefore not to restrict intravenous fluids during RALP.

A patient with undiagnosed mild mitral valve insufficiency reportedly experienced transitory exacerbation of that insufficiency during RALP [2]. A possible explanation for this is increased left ventricular after- and pre-loads; however, the degree of mitral insufficiency reverted to the initial status after the patient was repositioned [2].

In our study, the percutaneous rSO_2_ increased with head-down tilt, consistent with other studies [10, 13]. We also measured ScvO_2_, which did not change. These findings support the conclusion that the effects of steep head-down tilt and pneumoperitoneum on the cerebral circulation are relatively small. Using second-generation near infrared spectroscopy, Kalmar et al. recently reported that cerebral perfusion pressure was maintained above the lower threshold of cerebral autoregulation during RACP, likely because of simultaneous increases in MAP and central venous pressure [10].

### Conclusions

We found that LVEF decreased after steep head-down tilting and establishment of pneumoperitoneum for RALP, whereas CI and LVEDV measured by TEE, and CI measured by FloTrac^®^, did not change. These findings indicate that the steep head-down tilt and pneumoperitoneum during RALP did not greatly influence cardiac function in our cohort. Additionally, assessment of CI with TEE did not provide more useful information concerning cardiac function than was provided by FloTrac^®^ in these patients.

## Limitations

The limitations of our trial include its small size, the criteria for patient selection and the use of volume-controlled ventilation during RALP. Different settings for tidal volume and PEEP, or pressure-controlled ventilation, may have produced different results. In our institute, only patients who meet the criteria for ASA I–II undergo RALP. Because a pneumoperitoneum and steep Trendelenburg position are well tolerated by individuals with normal cardiac function, further clinical trials are required to evaluate those with cardiac or valve dysfunction or high pulmonary vascular pressure undergoing this procedure.

## Abbreviations

BP: blood pressure
CI: cardiac index
CO: cardiac output
CO_2_: carbon dioxide
LVEDP: left ventricular end-diastolic pressure
LVEDV: left ventricle end-diastolic volume
LVEF: left ventricular ejection fraction
MAP: mean arterial pressure
PEEP: positive end-expiratory pressure
RALP: robot-assisted laparoscopic prostatectomy
rSO2: regional saturation of oxygen
ScvO_2_: central venous blood oxygen saturation
TEE: transesophageal echocardiography
